# Effectiveness of vedolizumab to reduce bowel urgency in a Canadian real-world inflammatory bowel disease cohort

**DOI:** 10.1093/jcag/gwag031

**Published:** 2026-08-12

**Authors:** Parul Tandon, Yvette Leung, Ryan Ward, Abhinav Wadhwa, Jessica Weiss, Ruth Moulson, Christopher Pettengell, Robert Battat

**Affiliations:** Division of Gastroenterology and Hepatology, University Health Network, University of Toronto, Toronto, Ontario, M5T2S5, Canada; Division of Gastroenterology, University of British Columbia, Vancouver, British Columbia, V5Z 1M9, Canada; Takeda Canada Inc.Toronto, Ontario, M5H 4E3, Canada; Takeda Canada Inc.Toronto, Ontario, M5H 4E3, Canada; Pentavere Research Group Inc., Toronto, Ontario, M6G 1A1, Canada; Pentavere Research Group Inc., Toronto, Ontario, M6G 1A1, Canada; Pentavere Research Group Inc., Toronto, Ontario, M6G 1A1, Canada; Division of Gastroenterology, Centre Hospitalier de l’Université de Montréal, Montréal, Quebec H2X 0A9, Canada

**Keywords:** gastroenterology, real world evidence, bowel urgency, inflammatory bowel disease, vedolizumab, non-interventional

## Abstract

**Background and Aims:**

Bowel urgency is a burdensome symptom of inflammatory bowel disease (IBD) that greatly impacts quality of life. This study evaluated vedolizumab effectiveness on bowel urgency in Canadian patients with ulcerative colitis (UC) and Crohn’s disease (CD).

**Methods:**

Adults with moderately to severely active UC or CD with baseline Urgency Numeric Rating Scale (UNRS) scores enrolled in the Canadian Patient Support Program (July 2024–March 2025) were included. Bowel urgency and clinical endpoints were evaluated at 2, 6, and 14 weeks. Clinically meaningful improvement (CMI) was defined as UNRS change from baseline ≥3 among patients with baseline UNRS ≥3. UC clinical remission was defined as pM (partial Mayo) score <3. CD clinical response was defined as Harvey-Bradshaw Index (HBI) decrease ≥3 and ≥25% from baseline; remission as HBI <5.

**Results:**

Of 412 vedolizumab-treated patients (319 UC, 93 CD), most were bio-naïve (UC: 89%, CD: 63%) with median disease durations of 4 years (UC) and 7 years (CD). At baseline, 268 (84%) UC and 74 (80%) CD patients had UNRS ≥3. Median time to CMI was 6 weeks in both cohorts. By week 14, UC patients experienced a 5-point median UNRS reduction (80%) and CD patients a 4-point reduction (62%). Patients achieving CMI at weeks 6 and 14 were significantly more likely to achieve clinical remission. Week 2 urgency outcomes were significant predictors of later outcomes.

**Conclusions:**

Most UC and CD patients achieved CMI by 6 weeks. CMI was significantly associated with validated clinical outcomes, demonstrating real-world effectiveness of vedolizumab in reducing bowel urgency.

## Introduction

Inflammatory bowel disease (IBD) is a group of inflammatory conditions affecting the gastrointestinal tract, encompassing ulcerative colitis (UC) and Crohn’s disease (CD).^1^ Globally, IBD affects more than 5 million individuals, with Canada reporting among the highest incidence and prevalence rates.^1–3^ Due to its chronic nature, IBD significantly impacts patient quality of life and is commonly associated with psychological comorbidities including depression, anxiety, and fatigue.^4^^,^^5^ With the primary goal of IBD treatment being to induce and maintain remission while avoiding complications, current treatments often focus on clinical outcomes rather than on patient-reported outcomes that directly reflect daily functioning and quality of life.^6^^,^^7^

Bowel urgency, defined as the immediate or sudden need to have a bowel movement, is one of the most common and distressing symptoms in UC and CD.^8^ Patients consistently rank urgency control among their top treatment priorities, alongside surgery prevention and clinical remission, and persistent urgency despite clinical improvement is associated with lower treatment satisfaction and impaired quality of life.^9^^,^^10^ Conversely, achievement of bowel urgency remission is associated with significant quality-of-life improvement and supports long-term treatment persistence,^11^^,^^12^ a clinically important consideration given that fewer than half of IBD patients continue their initial biologic beyond 1 year.^13^ Bowel urgency is increasingly recognized as a meaningful patient-reported outcome, with recent Food and Drug Administration guidance recommending its evaluation in IBD,^14–16^ and evaluation via the Urgency Numeric Rating Scale (UNRS) has been content-validated in adults with UC.^14^ Despite this growing recognition, real-world data on bowel urgency response to currently available IBD therapies, particularly in patients receiving vedolizumab, remain limited.

Significant advances in IBD management have been driven by the development of targeted immunotherapies.^6^^,^^7^^,^^17^ One such example is vedolizumab (a gut-selective anti-lymphocyte trafficking drug indicated for the treatment of moderately to severely active UC and CD in adults).^18^^,^^19^ Vedolizumab is administered at a fixed dose of 300 mg by intravenous (IV) infusions at weeks 0, 2, and 6 during the induction period. Following induction, patients can receive maintenance IV treatment of the same dose every 8 weeks (Q8W) with the option of increased frequency to every 4 weeks (Q4W).^18^^,^^19^ Alternatively, patients can continue on subcutaneous maintenance vedolizumab by injection of 108 mg every 2 weeks (Q2W). Vedolizumab’s efficacy in inducing and maintaining clinical remission was established in the pivotal GEMINI 1 and GEMINI 2 phase 3 trials. ^20^^,^^21^ Subsequent real-world evidence, including national and international cohort studies and meta-analytic synthesis, has demonstrated comparable effectiveness in routine clinical practice.^22–24^ However, bowel urgency, as an increasingly recognized patient-reported outcome, remains less well explored among patients receiving vedolizumab.

This retrospective study leveraged data from the Takeda Canada Patient Support Program (PSP) to evaluate the real-world effectiveness of vedolizumab in reducing bowel urgency, as measured by UNRS score, among adults with moderately to severely active UC or CD. The study also assessed the relationship between changes in bowel urgency and other established clinical outcomes to support a comprehensive understanding of optimal IBD management.

## Methods

### Study design

This was a non-interventional, retrospective, longitudinal cohort study utilizing data from the Canadian Takeda PSP during the study period of July 2024 to March 2025. Adults (≥18 years) with moderately to severely active UC or CD (for CD, defined as a Harvey-Bradshaw Index [HBI] score of 8 or above, for UC, defined as a partial Mayo [pM] score of 5 or above) who initiated IV vedolizumab at a fixed dose of 300 mg and were enrolled in the PSP were eligible for inclusion. Patients were required to have provided written consent for secondary use of their data and have a baseline UNRS score recorded. The index date was defined as the date of vedolizumab initiation. Outcomes were evaluated at weeks 2, 6, and 14 post-index.

Baseline and outcome variables collected in this study included patient demographics (age and sex), disease characteristics (subtype, duration, previous biologic use, and disease activity scores), clinical biomarkers (C-reactive protein [CRP] and fecal calprotectin [FCP]), and vedolizumab treatment information (treatment start date, end date, infusion interval, and dosage). Disease scores included baseline and post-treatment assessments using UNRS scores prospectively collected for all patients, pM scores for patients with UC, and HBI scores for patients with CD. These variables informed descriptive summaries of the cohorts as well as clinical endpoints.

### Outcomes

Outcome measures included both bowel urgency-specific and clinical endpoints assessed at weeks 2, 6, and 14 following vedolizumab initiation. Bowel urgency clinically meaningful improvement (CMI) was defined as a reduction of ≥3 from baseline in UNRS score among patients with a baseline score of ≥3.^11^ Bowel urgency remission was defined as a UNRS score of 0 or 1.

For patients with UC, clinical response was defined as a decrease in pM score of ≥2 and ≥25% from baseline. Clinical remission was defined as a pM score <3. For patients with CD, clinical response was defined as a decrease in HBI score of ≥3 and ≥25% from baseline. Clinical remission was defined as an HBI score <5.

All outcomes were assessed at weeks 2, 6, and 14 among patients with baseline UNRS ≥3, HBI ≥8 (CD), and pM ≥5 (UC). Point and percentage reductions from baseline in UNRS score, time to CMI, associations between bowel urgency and clinical outcomes, and correlations between early and later bowel urgency outcomes are reported.

Analyses were performed separately among UC and CD patients with baseline UNRS scores ≥3. Patients were further stratified by biological experience (bio-naïve or bio-experienced), baseline disease severity (moderate or severe), and disease duration (<2 years from diagnosis or ≥2 years from diagnosis).

### Statistical analyses

Descriptive statistics were used to describe the demographic and clinical characteristics of the cohorts. Categorical variables were presented as counts and percentages. Continuous variables were summarized as mean and standard deviation (SD), median, minimum (min), and maximum (max) for continuous variables. Missing observations were reported as a separate category for each categorical, and continuous variable. The proportion of patients achieving bowel urgency and clinical outcomes was reported with associated binomial confidence intervals. Missing data were quantified in the descriptive analysis of the population, if any. Changes from baseline in bowel urgency outcomes were adjusted for patient demographics, using mixed-effects models for repeated measures, with missing at random assumption for handling missing data. Models included baseline UNRS score, HBI score, or pM score, in addition to baseline patient demographics. Time to events were analyzed using cumulative incidence curves. Associations between bowel urgency and clinical outcomes were explored using Fisher’s exact and chi-squared tests with significance specified by a 2-sided *P*-value of <.05. Correlation between early and late bowel urgency outcomes was explored using Spearman’s rank correlation coefficient for UNRS scores, and the phi coefficient was utilized for bowel CMI, and remission.

### Ethical statement

This study was conducted in accordance with the protocol approved by the Veritas Independent Review Board, the Declaration of Helsinki, ICH E6 Good Clinical Practice guidelines, Good Pharmacoepidemiology Practices, and applicable data privacy and regulatory requirements.

## Results

The study cohort consisted of 319 (89% bio-naïve) patients with UC and 93 (63% bio-naïve) patients with CD (Table 1), with 268 (84%) patients with UC, and 74 (80%) patients with CD, having a UNRS score of ≥3. Among patients with UC, median age was 45 years (range: 18-87 years) and 52% (*n* = 165/319) of patients were female, whereas among patients with CD, median age was 52 years (range: 19-76 years) and 66% of patients were female (*n* = 61/93; Table 1). Median disease duration prior to vedolizumab was 4 years for UC patients and 7 years for CD patients (Table 1). Median pM score at baseline among UC patients was 6 (range: 5-9) and median HBI score at baseline among CD patients was 9 (range: 8-24; Table 1).

**Table 1:** Baseline demographics and clinical characteristics of included patients with UC or CD treated with vedolizumab.

	Patients with UC (*N* = 319)	Patients with CD (*N* = 93)
**Age**		
**Mean (SD)**	47 (17)	50 (17)
**Median (IQR)**	45 (32-61)	52 (35-64)
**Range (min, max)**	18-87	19-76
**Sex (*n*, %)**		
** Male**	154 (48)	32 (34)
** Female**	165 (52)	61 (66)
**Disease duration, years**		
** Mean (SD)**	8 (10)	13 (14)
** Median**	4	7
** Range (min, max)**	0-38	0-50
** Missing**	14	7
**Previous biologic exposure (*n*, %)**		
** Bio-experienced**	36 (11)	34 (37)
** Bio-naïve**	283 (89)	59 (63)
**Urgency numerical rating scale at baseline**		
** Mean (SD)**	6 (3.1)	5.7 (3.1)
** Median**	7	7
** Range (min, max)**	0,10	0,10
**pM or HBI score at baseline**	**pM score**	**HBI score**
** Mean (SD)**	7 (1)	11 (3)
** Median**	6	9
** Range (min, max)**	5-9	8-24
**FCP (ug/g) at baseline**		
** Mean (SD)**	1299.4 (2011.0)	917.5 (1560.4)
** Median**	614.0	182.5
** Range (min, max)**	15.0-9311.0	43.0-5093.0
** Missing**	275	83
**CRP (mg/L) at baseline**		
** Mean (SD)**	30.9 (47.9)	5.1 (NA)
** Median**	5.0	5.1
** Range (min, max)**	1.6-86.2	5.1-5.1
** Missing**	316	92

Abbreviations: CD, Crohn’s disease; CRP, C-reactive protein; FCP, fecal calprotectin; HBI, Harvey-Bradshaw Index; SD, standard deviation; pM, partial Mayo; UC, Ulcerative colitis.

Among UC patients with a baseline UNRS score of ≥3, bowel urgency CMI rates increased from 45% (*n* = 114/253) at week 2 to 62% (*n* = 154/249) at week 6 and 67% (*n* = 86/128) at week 14, with corresponding bowel urgency remission rates of 26% (*n* = 67/253), 39% (*n* = 98/249), and 50% (*n* = 64/128) (Table 2). Clinical response and clinical remission rates rose in parallel, reaching 88% (*n* = 129/147) and 71% (*n* = 104/147), respectively, by week 14 (Table 2). Among CD patients with a baseline UNRS score of ≥3, bowel urgency CMI rates increased from 35% (*n* = 25/71) at week 2 to 53% (*n* = 39/73) at week 6 and 59% (*n* = 20/34) at week 14, with bowel urgency remission rates of 21% (*n* = 15/71), 32% (*n* = 23/73), and 38% (*n* = 13/34) across the same time points. CD clinical response reached 76% (*n* = 31/41) and clinical remission 56% (*n* = 23/41) by week 14 (Table 2).

**Table 2:** Bowel urgency and clinical outcomes at weeks 2, 6, and 14 after initiation of vedolizumab, among patients with UC or CD and baseline UNRS scores ≥3.

	Week 2		Week 6		Week 14	
	UC (*N* = 268)	CD (*N* = 74)	UC (*N* = 268)	CD (*N* = 74)	UC (*N* = 268)	CD (*N* = 74)
**Bowel urgency CMI, *n* (%) [95% CI]**						
** In bowel urgency CMI**	114 (45.1) [38.9-51.4]	25 (35.2) [24.5-47.5]	154 (61.8) [55.5-67.8]	39 (53.4) [41.4-65.0]	86 (67.2) [58.3-75.1]	20 (58.8) [40.8-74.9]
** Missing**	15	3	19	1	140	40
**Bowel urgency remission, *n* (%) [95% CI]**						
**In bowel urgency remission**	67 (26.5) [21.2-32.4]	15 (21.1) [12.7-32.7]	98 (39.4) [33.3-45.7]	23 (31.5) [21.4-43.6]	64 (50.0) [41.5-58.5]	13 (38.2) [22.7-56.4]
** Missing**	15	3	19	1	140	40
**Clinical remission, *n* (%)**						
** In clinical remission**	91 (34)	23 (31)	148 (56)	34 (46)	104 (71)	23 (56)
** Missing**	3	0	3	0	121	33
**Clinical response, *n* (%)**						
** In clinical response**	172 (65)	47 (64)	220 (83)	56 (76)	129 (88)	31 (76)
** Missing**	3	0	3	0	121	33

Abbreviations: CD, Crohn’s disease; CI, confidence interval; CMI, clinically meaningful improvement; UC, ulcerative colitis; UNRS, Urgency Numeric Rating Scale.

Ulcerative colitis patients with a UNRS score of **≥**3 experienced a median reduction of 2 points from baseline in UNRS scores by week 2, 3 points by week 6, and 5 points by week 14, equating to an 80% reduction overall (Table S1). Meanwhile, CD patients experienced a 1-point median reduction from baseline in UNRS scores by week 2, followed by a 3-point reduction by week 6 and a 4-point reduction by week 14, equating to a 62% reduction overall (Table S1).

The median time to bowel urgency CMI was approximately 6 weeks among UC patients (Figure 1A) and also for CD patients (Figure 1B) with UNRS scores of **≥**3. Time to bowel urgency CMI was comparable between bio-naïve and bio-experienced groups by week 14 (Figure 1A and B).

**Figure 1:**
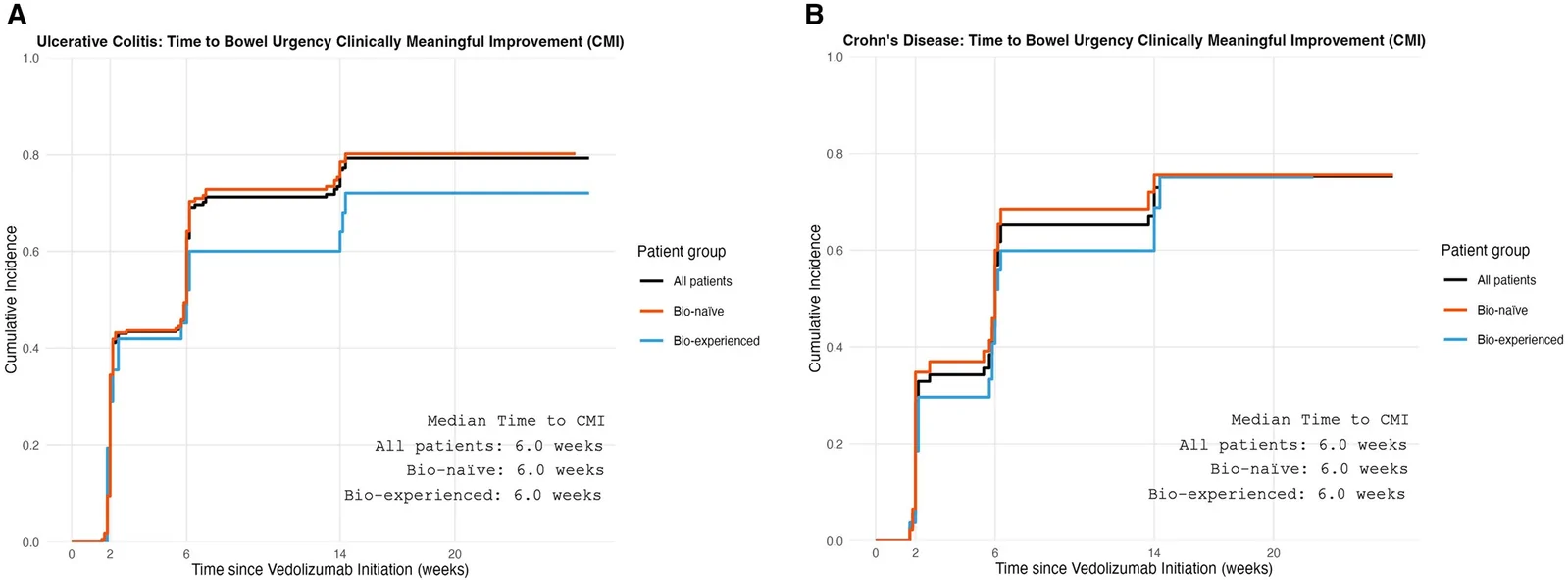
Time to bowel urgency CMI among patients with UC or CD with baseline UNRS scores ≥3. (A) UC time to bowel urgency CMI. (B) CD time to bowel urgency CMI. BU-CMI, Bowel urgency clinically meaningful improvement; CMI, clinically meaningful improvement; CD, Crohn’s disease; UNRS, Urgency Numeric Rating Scale; UC, ulcerative colitis.

Among both UC and CD patients there was a significant association between bowel urgency CMI and clinical outcomes at weeks 6 and 14 (Table S2). At week 14, UC patients experiencing bowel urgency CMI were more likely to be in clinical remission when compared to those not experiencing bowel urgency CMI (*n* = 75/86, 87% vs *n* = 12/42, 29%, *P* ≤ .001; Table S2), and more likely to be in clinical response (*n* = 84/86, 98% vs *n* = 27/42, 64%, *P* ≤ .001; Table S2). At week 14, CD patients experiencing bowel urgency CMI were more likely to be in clinical remission when compared to those not experiencing bowel urgency CMI (*n* = 14/20, 70% vs *n* = 3/14, 21%, *P* = .005; Table S2), and more likely to be in response as well (*n* = 18/20, 90% vs *n* = 6/14, 43%, *P* = .006; Table S2).

Lastly, among both UC and CD patients, early bowel urgency scores were positively and significantly associated with later bowel urgency scores (Table 3). UC patients who experienced bowel urgency CMI at week 2 were significantly more likely to experience bowel urgency CMI at week 14 (*P* < .004; Table 3).

**Table 3:** Correlations between early bowel urgency and later bowel urgency among patients with UC and CD.

UC	Correlations between weeks 2 and 6 (*N* = 240)		Correlations between weeks 2 and 14 (*N* = 125)	
	Spearman’s rank/phi coefficient	*P*-value	Spearman’s rank/phi coefficient	*P*-value
**UNRS score**	0.452	**<.001**	0.300	**<.001**
**Bowel urgency CMI**	0.390	**<.001**	0.255	**.004**
**Bowel urgency remission**	0.371	**<.001**	0.122	**.174**

CD	Correlations between weeks 2 and 6 (*N* = 71)		Correlations between weeks 2 and 14 (*N* = 34)	
	Spearman’s rank/phi coefficient	*P*-value	Spearman’s rank/phi coefficient	*P*-value
**UNRS score**	0.451	**<.001**	0.349	**.043**
**Bowel urgency CMI**	0.273	**.021**	0.243	**.167**
**Bowel urgency remission**	0.325	**.006**	0.048	**.786**

Abbreviations: CD, Crohn’s disease; CMI, clinically meaningful improvement; UC, ulcerative colitis. Bold *P*-values indicate statistical significance (*P* < .05)

## Discussion

This was a retrospective, non-interventional cohort study of 412 patients with UC or CD receiving IV vedolizumab and prospectively followed as part of the Canadian Takeda PSP. It describes the real-world effectiveness of vedolizumab in improving bowel urgency as an important patient-reported outcome, and associations between bowel urgency and existing clinical outcomes in IBD management.

This real-world study describes vedolizumab effectiveness in reducing bowel urgency over 14 weeks in a nationwide cohort of patients with moderate to severe UC and CD in routine clinical practice. Through vedolizumab treatment, over half of patients with CD and UC achieved CMI as early as week 6, with incremental improvements through week 14. These findings parallel data from controlled trial settings. In LUCENT-1/LUCENT-2 (UC), patients receiving mirikizumab experienced greater bowel urgency improvement than placebo at weeks 12 and 52 across baseline urgency severity strata.^14^ More recently, the VIVID-1 trial reported on bowel urgency in CD: mirikizumab-treated participants achieved significantly higher rates of bowel urgency CMI and remission at weeks 12 and 52 versus placebo, with CMI at week 12 associated with better short- and longer-term clinical and endoscopic outcomes.^25^ Our real-world findings extend the evidence base to vedolizumab—a mechanistically distinct (gut-selective α4β7) agent.

Previous studies have established the UNRS as a robust, content-valid, and reliable tool for assessing bowel urgency in adults with UC, and is increasingly recognized as a meaningful patient-reported outcome measure for capturing treatment effects in clinical trials involving patients with moderately to severely active disease.^14–16^ The present study demonstrates these improvements translate to routine practice; both UC and CD patients achieved a >60% reduction in UNRS scores by week 14, underscoring vedolizumab’s effectiveness in alleviating bowel urgency.

Bowel urgency in CD is mechanistically more heterogeneous than in UC. Whereas urgency in UC reflects primarily rectal and distal colonic inflammation, urgency in CD may arise through multiple pathways including colonic inflammation (L2/L3 disease), accelerated small-bowel transit and bile acid malabsorption in ileal disease, visceral hypersensitivity, and post-inflammatory functional changes persisting despite mucosal healing. Vedolizumab’s α4β7-mediated gut-selective mechanism could differentially benefit patients with colonic CD; however, without disease location data we cannot test this hypothesis. The proportion of UC patients achieving CMI at week 14 (67%) exceeded CD patients (59%), and the median UNRS reduction was greater in UC (−5) than CD (−4), consistent with a colonic-predominant mechanism of effect.

Our clinical outcomes are broadly consistent with the published real-world vedolizumab evidence base. Among comparable week-14 induction cohorts, the French OBSERV-IBD/GETAID cohort (*n* = 294) reported 36% steroid-free clinical remission and 50% response in UC, and 31% steroid-free remission and 51% response in CD^24^; a meta-analysis of vedolizumab real-world studies reported pooled week-14 remission rates of approximately 32% in UC and 30% in CD^23^; and the prospective Greek VEDO-IBD cohort of 96 UC patients reported 56.3% clinical remission and 76% response at week 14.^26^ Direct comparison is limited by heterogeneity in remission definitions, prior biologic exposure, and follow-up structure. Our cohort was restricted to patients with baseline UNRS ≥3—a symptomatically active subgroup—and was predominantly bio-naïve (89% UC, 63% CD). A previous Canadian analysis from the same PSP data source has likewise demonstrated meaningful response to vedolizumab across UC and CD.^22^ To our knowledge, the present study is among the first real-world Canadian analyses to quantify the timing and magnitude of bowel urgency response specifically, and to demonstrate that early (week 2) urgency improvements predict sustained week-14 outcomes.

Importantly, in this real-world cohort, bowel urgency CMI was strongly associated with improved clinical outcomes. At week 14, patients who achieved bowel urgency CMI were significantly more likely to be in clinical remission or response. Among UC patients, remission rates were 87% in those with bowel urgency CMI versus 29% in those without. In CD patients, rates were 70% versus 21% respectively. These associations were consistent with findings for clinical response. These findings suggest that vedolizumab is not only effective at improving existing clinical outcomes, as previously reported, but also improves symptom-specific outcomes that reflect patient experiences and have important implications for overall quality of life.^20–23^ Finally, early improvements in bowel urgency, as early as week 2 post-treatment, were predictive of sustained improvement through week 14, particularly among UC patients. This highlights the potential role of early symptom response as an indicator of longer-term benefit and supports its utility for early treatment monitoring and clinical decision-making.

Several limitations should be considered when interpreting these findings:

### Data limitations

The Takeda Canada PSP dataset is designed primarily to support patient access and reimbursement and therefore does not capture certain aspects of disease or treatment outside that scope; for example, disease location in CD or disease extension in UC, precluding correlation of bowel urgency findings with anatomical disease distribution. Objective markers of disease activity were not consistently captured; endoscopic, ultrasonographic data and biochemical markers (CRP, FCP) were available for only a minority of patients. Beyond UNRS, complementary patient-reported outcome measures such as Patient-Reported Outcomes Measurement Information System (PROMIS) fatigue or anxiety inventories were not collected. This effectiveness assessment relies predominantly on HBI, pM, and the patient-reported UNRS-validated and clinically meaningful instruments, but symptom-based rather than objective. Discordance between symptomatic improvement and mucosal or biochemical disease activity is well-recognized in IBD, and findings should be interpreted as reflecting symptomatic response rather than confirmed inflammatory disease control.

Nonetheless, UNRS continue to gain recognition as an essential component of clinical research and real-world evidence. Inclusion of patient-reported outcome measures (PROMs) like UNRS helps better align study designs with the principles of the Food and Drug Administration’s Patient Focused Drug Development initiative to enhance patient-centred care.^27^

### Design limitations

This was a retrospective, non-interventional cohort study with no concurrent comparator group; only differences between subgroups within the cohort were assessed. Causal inferences regarding vedolizumab’s specific contribution to observed improvements cannot be drawn.

### Interpretive limitations

Concomitant therapies, including corticosteroids, oral and rectal 5-ASA, and immunomodulators, were not captured in the PSP and could not be characterized in this cohort. Topical 5-ASA and short courses of corticosteroids may contribute to early symptomatic improvement, and a contribution from adjunctive therapy to the rapid week-2 response observed in UC cannot be excluded. While vedolizumab’s induction efficacy in randomized trials supports a timely treatment effect, the present analysis cannot disentangle the relative contributions of vedolizumab and concurrent therapies to early bowel urgency outcomes. The analysis was scoped to include data from weeks 2 to 14, when bowel urgency is rapidly modifiable; future work should assess the durability of bowel urgency response over longer-term IV maintenance.

A strength of this study is its use of nationwide real-world data, ensuring broad representativeness of the IBD population in Canada, where vedolizumab access through insurance and compassionate use programs further supports generalizability.

Bowel urgency remains one of the most common and burdensome symptoms for individuals living with IBD. In this real-world study, most patients achieved bowel urgency CMI within 6 weeks of IV vedolizumab, and CMI was significantly associated with validated clinical outcomes. This study adds to the evidence supporting vedolizumab’s role in improving both disease control and symptom-related quality of life in moderate to severe UC and CD. Incorporating bowel urgency assessment into routine IBD care may better align therapeutic decision-making with patient priorities, and early symptom response may be as informative as traditional clinical endpoints for guiding long-term management.

## Supplementary Material

gwag031_Supplementary_Data

## Data Availability

The data underlying this article cannot be shared publicly due to the privacy of individuals that participated in the nation-wide patient support program, developed by Takeda Canada Inc. The data will be shared on reasonable request to the corresponding author.
