# Supplementary material for: Effectiveness of vedolizumab to reduce bowel urgency in a Canadian real-world inflammatory bowel disease cohort
Source: J Can Assoc Gastroenterol. 2026 Aug 12;9(5):320–6. doi: 10.1093/jcag/gwag031 (PMC13639363; doi:10.1093/jcag/gwag031)
Supplement: Supplementary file 1 [file gwag031_supplementary_data.zip › coi_disclosures_all_authors.pdf]

ICMJE DISCLOSURE FORM

Date: 9-june-2026
Your Name: Robert Battat
Manuscript Title: Effectiveness of Vedolizumab to Reduce Bowel Urgency in a Canadian Real World Inflammatory Bowel Disease Cohort
Manuscript number (if known): JCAG-2025-0044

In the interest of transparency, we ask you to disclose all relationships/activities/interests listed below that are related to the content of your manuscript. "Related" means any relation with for-profit or not-for-profit third parties whose interests may be affected by the content of the manuscript. Disclosure represents a commitment to transparency and does not necessarily indicate a bias. If you are in doubt about whether to list a relationship/activity/interest, it is preferable that you do so.

The following questions apply to the author's relationships/activities/interests as they relate to the current manuscript only.

The author's relationships/activities/interests should be defined broadly. For example, if your manuscript pertains to the epidemiology of hypertension, you should declare all relationships with manufacturers of antihypertensive medication, even if that medication is not mentioned in the manuscript.

In item #1 below, report all support for the work reported in this manuscript without time limit. For all other items, the time frame for disclosure is the past 36 months.

		**Name all entities with whom you have this relationship or indicate none (add rows as needed)**	**Specifications/Comments (e.g., if payments were made to you or to your institution)**
**Time frame: Since the initial planning of the work**			
1	All support for the present manuscript (e.g., funding, provision of study materials, medical writing, article processing charges, etc.) **No time limit for this item.**	Consulting with Takeda	
**Time frame: past 36 months**			
2	Grants or contracts from any entity (if not indicated in item #1 above).	Research support from Celltrion	
3	Royalties or licenses	____ None	

4	Consulting fees	____ Bristol Myers Squibb, Johnson and Johnson Innovative Medicine, AbbVie, Takeda, Ferring, Celltrion, Pfizer, Merck, Eli Lilly, GSK	
5	Payment or honoraria for lectures, presentations, speakers bureaus, manuscript writing or educational events	____ Celltrion, AbbVie, Pfizer, Janssen, Bristol Myers Squibb, Takeda, Merck, Eli Lilly	
6	Payment for expert testimony	____ None	
7	Support for attending meetings and/or travel	____ Pfizer, Celltrion, Johnson and Johnson Innovative Medicine, Abbvie	
8	Patents planned, issued or pending	____ None	
9	Participation on a Data Safety Monitoring Board or Advisory Board	____ Celltrion, AbbVie, Pfizer, Janssen, Bristol Myers Squibb, Takeda, Merck, Eli Lilly	
10	Leadership or fiduciary role in other board, society, committee or advocacy group, paid or unpaid	____ None	
11	Stock or stock options	____ None	
12	Receipt of equipment, materials, drugs, medical writing, gifts or other services	____ None	

13	Other financial or non-financial interests	____ None	

Please place an "X" next to the following statement to indicate your agreement:

X I certify that I have answered every question and have not altered the wording of any of the questions on this form.

ICMJE DISCLOSURE FORM

Date: June 11, 2026
Your Name: Yvette Leung
Manuscript Title: Effectiveness of Vedolizumab to Reduce Bowel Urgency in a Canadian Real World Inflammatory Bowel Disease Cohort
Manuscript number (if known): JCAG-2025-0044

In the interest of transparency, we ask you to disclose all relationships/activities/interests listed below that are related to the content of your manuscript. "Related" means any relation with for-profit or not-for-profit third parties whose interests may be affected by the content of the manuscript. Disclosure represents a commitment to transparency and does not necessarily indicate a bias. If you are in doubt about whether to list a relationship/activity/interest, it is preferable that you do so.

The following questions apply to the author's relationships/activities/interests as they relate to the current manuscript only.

The author's relationships/activities/interests should be defined broadly. For example, if your manuscript pertains to the epidemiology of hypertension, you should declare all relationships with manufacturers of antihypertensive medication, even if that medication is not mentioned in the manuscript.

In item #1 below, report all support for the work reported in this manuscript without time limit. For all other items, the time frame for disclosure is the past 36 months.

		**Name all entities with whom you have this relationship or indicate none (add rows as needed)**	**Specifications/Comments (e.g., if payments were made to you or to your institution)**
**Time frame: Since the initial planning of the work**			
1	All support for the present manuscript (e.g., funding, provision of study materials, medical writing, article processing charges, etc.) **No time limit for this item.**	Consulting with Takeda	
**Time frame: past 36 months**			
2	Grants or contracts from any entity (if not indicated in item #1 above).	____ None	
3	Royalties or licenses	____ None	

4	Consulting fees	Janssen, Abbvie, Takeda, Celltrion, Pfizer, Eli Lilly	
5	Payment or honoraria for lectures, presentations, speakers bureaus, manuscript writing or educational events	Janssen, Abbvie, Takeda, Celltrion, Pfizer, Eli Lilly	
6	Payment for expert testimony	____ None	
7	Support for attending meetings and/or travel	Janssen, Abbvie, Takeda, Celltrion, Pfizer, Eli Lilly	
8	Patents planned, issued or pending	____ None	
9	Participation on a Data Safety Monitoring Board or Advisory Board	____ None	
10	Leadership or fiduciary role in other board, society, committee or advocacy group, paid or unpaid	____ None	
11	Stock or stock options	____ None	
12	Receipt of equipment, materials, drugs, medical writing, gifts or other services	____ None	
13	Other financial or non-financial interests	____ None	

Please place an "X" next to the following statement to indicate your agreement:

X I certify that I have answered every question and have not altered the wording of any of the questions on this form.

ICMJE DISCLOSURE FORM

Date: 9/5/2025

Your Name: Ruth Moulson

Manuscript Title: **Effectiveness of Vedolizumab to Reduce Bowel Urgency in a Canadian Real World Inflammatory Bowel Disease Cohort**

Manuscript Number (if known): JCAG-2025-0044

In the interest of transparency, we ask you to disclose all relationships/activities/interests listed below that are related to the content of your manuscript. "Related" means any relation with for-profit or not-for-profit third parties whose interests may be affected by the content of the manuscript. Disclosure represents a commitment to transparency and does not necessarily indicate a bias. If you are in doubt about whether to list a relationship/activity/interest, it is preferable that you do so.

The author's relationships/activities/interests should be defined broadly. For example, if your manuscript pertains to the epidemiology of hypertension, you should declare all relationships with manufacturers of antihypertensive medication, even if that medication is not mentioned in the manuscript.

In item #1 below, report all support for the work reported in this manuscript without time limit. For all other items, the time frame for disclosure is the past 36 months.

		Name all entities with whom you have this relationship or indicate none (add rows as needed)	Specifications/Comments (e.g., if payments were made to you or to your institution)
**Time frame: Since the initial planning of the work**			
**1**	All support for the present manuscript (e.g., funding, provision of study materials, medical writing, article processing charges, etc.) **No time limit for this item.**	☐ **None**	
			Click the tab key to add additional rows.
**Time frame: past 36 months**			
**2**	Grants or contracts from any entity (if not indicated in item #1 above).	☐ **None**	

		Name all entities with whom you have this relationship or indicate none (add rows as needed)	Specifications/Comments (e.g., if payments were made to you or to your institution)
3	Royalties or licenses	☐ None	
4	Consulting fees	☐ None	
5	Payment or honoraria for lectures, presentations, speakers bureaus, manuscript writing or educational events	☐ None	
6	Payment for expert testimony	☐ None	
7	Support for attending meetings and/or travel	☐ None	
8	Patents planned, issued or pending	☐ None	
9	Participation on a Data Safety Monitoring Board or Advisory Board	☐ None	
10	Leadership or fiduciary role in other board,	☐ None	

		Name all entities with whom you have this relationship or indicate none (add rows as needed)	Specifications/Comments (e.g., if payments were made to you or to your institution)
	society, committee or advocacy group, paid or unpaid		
**11**	Stock or stock options	☐ **None**	
**12**	Receipt of equipment, materials, drugs, medical writing, gifts or other services	☐ **None**	
**13**	Other financial or non-financial interests	☐ **None**	

Please place an "X" next to the following statement to indicate your agreement:

☒ I certify that I have answered every question and have not altered the wording of any of the questions on this form.

ICMJE DISCLOSURE FORM

Date: 9/5/2026
Your Name: Christopher Pettengell
Manuscript Title: Effectiveness of Vedolizumab to Reduce Bowel Urgency in a Canadian Real World Inflammatory Bowel Disease Cohort
Manuscript number (if known): JCAG-2025-0044

In the interest of transparency, we ask you to disclose all relationships/activities/interests listed below that are related to the content of your manuscript. "Related" means any relation with for-profit or not-for-profit third parties whose interests may be affected by the content of the manuscript. Disclosure represents a commitment to transparency and does not necessarily indicate a bias. If you are in doubt about whether to list a relationship/activity/interest, it is preferable that you do so.

The following questions apply to the author's relationships/activities/interests as they relate to the current manuscript only.

The author's relationships/activities/interests should be defined broadly. For example, if your manuscript pertains to the epidemiology of hypertension, you should declare all relationships with manufacturers of antihypertensive medication, even if that medication is not mentioned in the manuscript.

In item #1 below, report all support for the work reported in this manuscript without time limit. For all other items, the time frame for disclosure is the past 36 months.

		**Name all entities with whom you have this relationship or indicate none (add rows as needed)**	**Specifications/Comments (e.g., if payments were made to you or to your institution)**
		**Time frame: Since the initial planning of the work**	
1	All support for the present manuscript (e.g., funding, provision of study materials, medical writing, article processing charges, etc.) **No time limit for this item.**	____ None	
		**Time frame: past 36 months**	
2	Grants or contracts from any entity (if not indicated in item #1 above).	____ None	
3	Royalties or licenses	____ None	

4	Consulting fees	____ None	
5	Payment or honoraria for lectures, presentations, speakers bureaus, manuscript writing or educational events	____ None	
6	Payment for expert testimony	____ None	
7	Support for attending meetings and/or travel	____ None	
8	Patents planned, issued or pending	____ None	
9	Participation on a Data Safety Monitoring Board or Advisory Board	____ None	
10	Leadership or fiduciary role in other board, society, committee or advocacy group, paid or unpaid	____ None	
11	Stock or stock options	____ None	
12	Receipt of equipment, materials, drugs, medical writing, gifts or other services	____ None	
13	Other financial or non-financial interests	____ None	

Please place an "X" next to the following statement to indicate your agreement:

X I certify that I have answered every question and have not altered the wording of any of the questions on this form.

ICMJE DISCLOSURE FORM

Date: May 21, 2026
Your Name: Parul Tandon
Manuscript Title: Effectiveness of Vedolizumab to Reduce Bowel Urgency in a Canadian Real World Inflammatory Bowel Disease Cohort
Manuscript number (if known): JCAG-2025-0044

In the interest of transparency, we ask you to disclose all relationships/activities/interests listed below that are related to the content of your manuscript. "Related" means any relation with for-profit or not-for-profit third parties whose interests may be affected by the content of the manuscript. Disclosure represents a commitment to transparency and does not necessarily indicate a bias. If you are in doubt about whether to list a relationship/activity/interest, it is preferable that you do so.

The following questions apply to the author's relationships/activities/interests as they relate to the current manuscript only.

The author's relationships/activities/interests should be defined broadly. For example, if your manuscript pertains to the epidemiology of hypertension, you should declare all relationships with manufacturers of antihypertensive medication, even if that medication is not mentioned in the manuscript.

In item #1 below, report all support for the work reported in this manuscript without time limit. For all other items, the time frame for disclosure is the past 36 months.

		**Name all entities with whom you have this relationship or indicate none (add rows as needed)**	**Specifications/Comments (e.g., if payments were made to you or to your institution)**
		**Time frame: Since the initial planning of the work**	
1	All support for the present manuscript (e.g., funding, provision of study materials, medical writing, article processing charges, etc.) **No time limit for this item.**	____ None	
		Takeda	Medical writing; No payment made to me directly
		**Time frame: past 36 months**	
2	Grants or contracts from any entity (if not indicated in item #1 above).	__xx__ None	
3	Royalties or licenses	___x_ None	

4	Consulting fees	____ None<br>Abbvie, Takeda, J+J	
5	Payment or honoraria for lectures, presentations, speakers bureaus, manuscript writing or educational events	____ None<br>Abbvie, Takeda, J+J, PEndopharm, Avir Pharma, Organon	
6	Payment for expert testimony	__x__ None	
7	Support for attending meetings and/or travel	__x__ None	
8	Patents planned, issued or pending	__x__ None	
9	Participation on a Data Safety Monitoring Board or Advisory Board	____ None	
10	Leadership or fiduciary role in other board, society, committee or advocacy group, paid or unpaid	__x__ None	
11	Stock or stock options	__x__ None	
12	Receipt of equipment, materials, drugs, medical writing, gifts or other services	__x__ None	
13	Other financial or non-financial interests	__x__ None	

Please place an "X" next to the following statement to indicate your agreement:

x I certify that I have answered every question and have not altered the wording of any of the questions on this form.

ICMJE DISCLOSURE FORM

**Date:**	6/3/2026
**Your Name:**	Abhinav Wadhwa
**Manuscript Title:**	Effectiveness of Vedolizumab to Reduce Bowel Urgency in a Canadian Real World Inflammatory Bowel Disease Cohort t
**Manuscript Number (if known):**	JCAG-2025-0044

In the interest of transparency, we ask you to disclose all relationships/activities/interests listed below that are related to the content of your manuscript. "Related" means any relation with for-profit or not-for-profit third parties whose interests may be affected by the content of the manuscript. Disclosure represents a commitment to transparency and does not necessarily indicate a bias. If you are in doubt about whether to list a relationship/activity/interest, it is preferable that you do so.

The author's relationships/activities/interests should be defined broadly. For example, if your manuscript pertains to the epidemiology of hypertension, you should declare all relationships with manufacturers of antihypertensive medication, even if that medication is not mentioned in the manuscript.

In item #1 below, report all support for the work reported in this manuscript without time limit. For all other items, the time frame for disclosure is the past 36 months.

		**Name all entities with whom you have this relationship or indicate none (add rows as needed)**	**Specifications/Comments (e.g., if payments were made to you or to your institution)**
**Time frame: Since the initial planning of the work**			
**1**	All support for the present manuscript (e.g., funding, provision of study materials, medical writing, article processing charges, etc.) **No time limit for this item.**	☐ **None**	
		This work was funded by Takeda Canada Inc. and conducted by Pentavere Research Group. Medical writing support was provided by Andrew Ho of Pentavere Research Group	
			Click the tab key to add additional rows.
**Time frame: past 36 months**			
**2**	Grants or contracts from any entity (if not indicated in item #1 above).	☒ **None**	

		Name all entities with whom you have this relationship or indicate none (add rows as needed)	Specifications/Comments (e.g., if payments were made to you or to your institution)
3	Royalties or licenses	☒ None	
4	Consulting fees	☒ None	
5	Payment or honoraria for lectures, presentations, speakers bureaus, manuscript writing or educational events	☒ None	
6	Payment for expert testimony	☒ None	
7	Support for attending meetings and/or travel	☒ None	
8	Patents planned, issued or pending	☒ None	
9	Participation on a Data Safety Monitoring Board or Advisory Board	☒ None	
10	Leadership or fiduciary role in other board,	☒ None	

		Name all entities with whom you have this relationship or indicate none (add rows as needed)	Specifications/Comments (e.g., if payments were made to you or to your institution)
	society, committee or advocacy group, paid or unpaid		
**11**	Stock or stock options	☒ **None**	
**12**	Receipt of equipment, materials, drugs, medical writing, gifts or other services	☒ **None**	
**13**	Other financial or non-financial interests	☐ **None**	
		Abhinav Wadhwa is an employee of Takeda Canada Inc.	

Please place an "X" next to the following statement to indicate your agreement:

☒ I certify that I have answered every question and have not altered the wording of any of the questions on this form.

ICMJE DISCLOSURE FORM

**Date:**	6/3/2026
**Your Name:**	Ryan Ward
**Manuscript Title:**	Effectiveness of Vedolizumab to Reduce Bowel Urgency in a Canadian Real World Inflammatory Bowel Disease Cohort
**Manuscript Number (if known):**	JCAG-2025-0044

In the interest of transparency, we ask you to disclose all relationships/activities/interests listed below that are related to the content of your manuscript. "Related" means any relation with for-profit or not-for-profit third parties whose interests may be affected by the content of the manuscript. Disclosure represents a commitment to transparency and does not necessarily indicate a bias. If you are in doubt about whether to list a relationship/activity/interest, it is preferable that you do so.

The author's relationships/activities/interests should be defined broadly. For example, if your manuscript pertains to the epidemiology of hypertension, you should declare all relationships with manufacturers of antihypertensive medication, even if that medication is not mentioned in the manuscript.

In item #1 below, report all support for the work reported in this manuscript without time limit. For all other items, the time frame for disclosure is the past 36 months.

		**Name all entities with whom you have this relationship or indicate none (add rows as needed)**	**Specifications/Comments (e.g., if payments were made to you or to your institution)**
**Time frame: Since the initial planning of the work**			
**1**	All support for the present manuscript (e.g., funding, provision of study materials, medical writing, article processing charges, etc.) **No time limit for this item.**	☐ **None**	
		This work was funded by Takeda Canada Inc. and conducted by Pentavere Research Group. Medical writing support was provided by Andrew Ho of Pentavere Research Group	
			Click the tab key to add additional rows.
**Time frame: past 36 months**			
**2**	Grants or contracts from any entity (if not indicated in item #1 above).	☒ **None**	

		Name all entities with whom you have this relationship or indicate none (add rows as needed)	Specifications/Comments (e.g., if payments were made to you or to your institution)
3	Royalties or licenses	☒ None	
4	Consulting fees	☒ None	
5	Payment or honoraria for lectures, presentations, speakers bureaus, manuscript writing or educational events	☒ None	
6	Payment for expert testimony	☒ None	
7	Support for attending meetings and/or travel	☒ None	
8	Patents planned, issued or pending	☒ None	
9	Participation on a Data Safety Monitoring Board or Advisory Board	☒ None	
10	Leadership or fiduciary role in other board,	☒ None	

		Name all entities with whom you have this relationship or indicate none (add rows as needed)	Specifications/Comments (e.g., if payments were made to you or to your institution)
	society, committee or advocacy group, paid or unpaid		
**11**	Stock or stock options	☒ **None**	
**12**	Receipt of equipment, materials, drugs, medical writing, gifts or other services	☒ **None**	
**13**	Other financial or non-financial interests	☐ **None**	
		Ryan Ward is an employee of Takeda Canada Inc.	

Please place an "X" next to the following statement to indicate your agreement:

☒ I certify that I have answered every question and have not altered the wording of any of the questions on this form.

ICMJE DISCLOSURE FORM

**Date:**	9/5/2026
**Your Name:**	Jessica Weiss
**Manuscript Title:**	**Effectiveness of Vedolizumab to Reduce Bowel Urgency in a Canadian Real World Inflammatory Bowel Disease Cohort t**
**Manuscript Number (if known):**	JCAG-2025-0044

In the interest of transparency, we ask you to disclose all relationships/activities/interests listed below that are related to the content of your manuscript. "Related" means any relation with for-profit or not-for-profit third parties whose interests may be affected by the content of the manuscript. Disclosure represents a commitment to transparency and does not necessarily indicate a bias. If you are in doubt about whether to list a relationship/activity/interest, it is preferable that you do so.

The author's relationships/activities/interests should be defined broadly. For example, if your manuscript pertains to the epidemiology of hypertension, you should declare all relationships with manufacturers of antihypertensive medication, even if that medication is not mentioned in the manuscript.

In item #1 below, report all support for the work reported in this manuscript without time limit. For all other items, the time frame for disclosure is the past 36 months.

		**Name all entities with whom you have this relationship or indicate none (add rows as needed)**	**Specifications/Comments (e.g., if payments were made to you or to your institution)**
**Time frame: Since the initial planning of the work**			
**1**	All support for the present manuscript (e.g., funding, provision of study materials, medical writing, article processing charges, etc.) **No time limit for this item.**	☐ **None**	
		This work was funded by Takeda Canada Inc. and conducted by Pentavere Research Group. Medical writing support was provided by Ruth Moulson of Pentavere Research Group	
			Click the tab key to add additional rows.
**Time frame: past 36 months**			
**2**	Grants or contracts from any entity (if not indicated in item #1 above).	☒ **None**	

		Name all entities with whom you have this relationship or indicate none (add rows as needed)	Specifications/Comments (e.g., if payments were made to you or to your institution)
3	Royalties or licenses	☒ None	
4	Consulting fees	☒ None	
5	Payment or honoraria for lectures, presentations, speakers bureaus, manuscript writing or educational events	☒ None	
6	Payment for expert testimony	☒ None	
7	Support for attending meetings and/or travel	☒ None	
8	Patents planned, issued or pending	☒ None	
9	Participation on a Data Safety Monitoring Board or Advisory Board	☒ None	
10	Leadership or fiduciary role in other board,	☒ None	

		Name all entities with whom you have this relationship or indicate none (add rows as needed)	Specifications/Comments (e.g., if payments were made to you or to your institution)
	society, committee or advocacy group, paid or unpaid		
**11**	Stock or stock options	☒ **None**	
**12**	Receipt of equipment, materials, drugs, medical writing, gifts or other services	☒ **None**	
**13**	Other financial or non-financial interests	☐ **None**	
		Jessica Weiss is an employee of Pentavere Research Group.	

Please place an "X" next to the following statement to indicate your agreement:

☒ I certify that I have answered every question and have not altered the wording of any of the questions on this form.
