# Supplementary material for: Effectiveness of vedolizumab to reduce bowel urgency in a Canadian real-world inflammatory bowel disease cohort
Source: J Can Assoc Gastroenterol. 2026 Aug 12;9(5):320–6. doi: 10.1093/jcag/gwag031 (PMC13639363; doi:10.1093/jcag/gwag031)
Supplement: Supplementary file 1 [file gwag031_supplementary_data.zip › Vedolizumab Bowel Urgency Supplementary_Tables_JCAG_2025_0044.docx]

**Supplementary Table S1.** Change from baseline UNRS scores at weeks 2, 6, and 14 after vedolizumab initiation among patients with UC or CD with baseline UNRS scores ≥3

|  | **Patients with UC with baseline UNRS scores** ≥**3**  **(N=268)** | **Patients with CD with baseline UNRS scores** ≥**3**  **(N=74)** |
| --- | --- | --- |
| **Week 2 change from baseline UNRS** |  |  |
| Absolute change |  |  |
| Mean (SD) | -2 (3) | -2 (3) |
| Median (Q1, Q3) | -2 (-5, 0) | -1 (-4, 0) |
| Percentage change (%) |  |  |
| Mean (SD) | -34 (53) | -27 (49) |
| Median (Q1, Q3) | -30 (-80, 0) | -20 (-67, 0) |
| **Week 6 change from baseline UNRS** |  |  |
| Absolute change |  |  |
| Mean (SD) | -4 (4) | -3 (4) |
| Median (Q1, Q3) | -3 (-6, -1) | -3 (-5, -1) |
| Percentage change (%) |  |  |
| Mean (SD) | -50 (52) | -39 (54) |
| Median (Q1, Q3) | -56 (-100, -20) | -43 (-100, -10) |
| **Week 14 change from baseline UNRS** |  |  |
| Absolute change |  |  |
| Mean (SD) | -4 (4) | -3 (4) |
| Median (Q1, Q3) | -5 (-8, -1) | -4 (-6, 0) |
| Percentage change (%) |  |  |
| Mean (SD) | -54 (60) | -44 (55) |
| Median (Q1, Q3) | -80 (-100, -18) | -62 (-100, 0) |

CD: Crohn’s disease; UNRS: Urgency Numeric Rating Scale; Q1: First quartile; Q3: Third quartile; SD: standard deviation; UC: Ulcerative colitis

**Supplementary Table S2.** Associations between bowel urgency CMI and clinical remission and response among patients with UC or CD with baseline UNRS scores ≥3

|  | **Week 6** | | | **Week 14** | | |
| --- | --- | --- | --- | --- | --- | --- |
| **UC** | **In bowel urgency CMI (N=154)** | **Not in bowel urgency CMI (N=95)** | **p-value** | **In bowel urgency CMI (N=86)** | **Not in bowel urgency CMI (N=42)** | **p-value** |
| **Clinical remission (n, %)** |  |  | **< 0.001** |  |  | **< 0.001** |
| In clinical remission | 105 (68%) | 30 (32%) |  | 75 (87%) | 12 (29%) |  |
| Not in clinical remission | 49 (32%) | 65 (68%) |  | 11 (13%) | 30 (71%) |  |
| **Clinical response (n, %)** |  |  | **< 0.001** |  |  | **< 0.001** |
| In clinical response | 138 (90%) | 67 (71%) |  | 84 (98%) | 27 (64%) |  |
| Not in clinical response | 16 (10%) | 28 (29%) |  | 2 (2%) | 15 (36%) |  |
| **CD** | **In bowel urgency CMI N=39** | **Not in bowel urgency CMI N=34** | **p-value** | **In bowel urgency CMI N=20** | **Not in bowel urgency CMI N=14** | **p-value** |
| **Clinical remission (n, %)** |  |  | **0.003** |  |  | **0.005** |
| In clinical remission | 24 (62%) | 9 (26%) |  | 14 (70%) | 3 (21%) |  |
| Not in clinical remission | 15 (38%) | 25 (74%) |  | 6 (30%) | 11 (79%) |  |
| **Clinical response (n, %)** |  |  | **< 0.001** |  |  | **0.006** |
| In clinical response | 36 (92%) | 19 (56%) |  | 18 (90%) | 6 (43%) |  |
| Not in clinical response | 3 (8%) | 15 (44%) |  | 2 (10%) | 8 (57%) |  |

CD: Crohn’s disease; CMI: Clinically meaningful improvement; UNRS: Urgency Numeric Rating Scale; UC: Ulcerative colitis
